# Central Effects of Botulinum Neurotoxin—Evidence from Human Studies

**DOI:** 10.3390/toxins11010021

**Published:** 2019-01-06

**Authors:** David Weise, Christopher M. Weise, Markus Naumann

**Affiliations:** 1 Department of Neurology, Asklepios Fachklinikum Stadtroda, Bahnhofstr. 1a, D-07646 Stadtroda, Germany; 2 Department of Neurology, University of Leipzig, Liebigstr. 20, D-04103 Leipzig, Germany; christopher.weise@medizin.uni-leipzig.de; 3 Department of Neurology and Clinical Neurophysiology, Augsburg University, Stenglinstrasse 2, D-86156 Augsburg, Germany

**Keywords:** Botulinum neurotoxin, central nervous system, spasticity, dystonia, human studies

## Abstract

For more than three decades, Botulinum neurotoxin (BoNT) has been used to treat a variety of clinical conditions such as spastic or dystonic disorders by inducing a temporary paralysis of the injected muscle as the desired clinical effect. BoNT is known to primarily act at the neuromuscular junction resulting in a biochemical denervation of the treated muscle. However, recent evidence suggests that BoNT’s pharmacological properties may not only be limited to local muscular denervation at the injection site but may also include additional central effects. In this review, we report and discuss the current evidence for BoNT’s central effects based on clinical observations, neurophysiological investigations and neuroimaging studies in humans. Collectively, these data strongly point to indirect mechanisms via changes to sensory afferents that may be primarily responsible for the marked plastic effects of BoNT on the central nervous system. Importantly, BoNT-related central effects and consecutive modulation and/or reorganization of the brain may not solely be considered “side-effects” but rather an additional therapeutic impact responsible for a number of clinical observations that cannot be explained by merely peripheral actions.

## 1. Introduction

Botulinum neurotoxin type A (BoNT) is used in a variety of clinical conditions such as spastic or dystonic disorders [1]. BoNT reduces muscle hyperactivity via its action at the neuromuscular junction. It does this by binding and internalization by the presynaptic cholinergic neuron. It cleaves SNARE (soluble N-ethylmaleimide-sensitive-factor attachment receptor) complex proteins and blocks acetylcholine release at the neuromuscular junction, thus resulting in a transient denervation and weakening of muscle contractions responsible for excessive involuntary movements. Although it is widely accepted that its therapeutic effects are restricted to the peripheral nervous system, additional actions at distant sites and central effects are also presumed. These central effects may be the consequence of hematogenic spread, a retrograde neural transport of BoNT to the central nervous system (CNS) or an indirect action due to denervation and changes of afferent input resulting in the plastic reorganization of the CNS [2].

Several early animal studies provide evidence for a retrograde transport of BoNT, similar to the well-known retrograde transport of tetanus toxin [3,4], but this has been highly debated. Following intramuscular injection of radiolabeled BoNT in the cat gastrocnemius muscle, radioactivity could be found successively in the sciatic nerve, the ipsilateral spinal ventral roots and the spinal cord with a distal-proximal gradient [5]. In parallel, functional changes on parts of the soma membrane of the alpha-motoneuron have been suggested in a follow-up neurophysiological study [6]; however, there was no proof for a distant active, catalytic effect of BoNT. In a more recent in vivo rodent study, Antonucci and colleagues showed that BoNT acted at facial nucleus neurons after injection in the whisker muscles [7]. Consistently, this and other groups were able to detect cleaved SNAP25 (synaptosomal nerve-associated protein 25) at distant cells, upstream from the initial uptake neurons, indicating catalytic action following retrograde interneuronal transport via transcystosis [7,8,9]. Functional evidence for bilateral muscle relaxation was observed after unilateral injection of commercially used BoNT in the rat paw. Here, BoNT arrived at the contralateral muscle to almost similar extents via neural pathways and the hematogenic route, suggesting transport within neuronal networks as an additional mechanism for BoNT’s action at distant sites [9].

In this review, we focus on the main human experimental and clinical studies providing information on the central action of BoNT. Central effects are discussed based on clinical observations as well as neurophysiological and imaging studies.

## 2. Clinical Evidence

Experience from clinical routine already suggests that BoNT-related muscle weakening may not exclusively be the consequence of BoNT’s action at the neuromuscular junction, but also at the spinal and supraspinal levels. In dystonia, clinical improvement without or with little weakness is frequently observed, whereas other patients do not improve despite relevant muscle weakness [10,11], thus only experiencing side effects without clinical benefit. Other patients with dystonia or spasticity may request retreatment with BoNT, irrespective of residual neuromuscular blockade [12]. Conversely, patients may benefit from BoNT treatment regardless of comparably little neuromuscular blockade [13]. Although acetylcholine release is blocked at the neuromuscular junction for around 12 weeks, some patients experience disproportionate muscle weakness or clinical benefit for many months, exceeding the average duration of peripheral chemo-denervation [14,15]. Accordingly, the peripheral paralyzing effect cannot be the only effect of BoNT [16]. Furthermore, in our clinical practice, some patients, particularly in dystonia, but also spastic syndromes, benefit considerably from BoNT treatment despite the use of relatively small or even “homeopathic” doses. Some patients are also known as “golden responders”, with long-lasting clinical benefits following a single or only few injections [17] which can only be explained by an indirect central effect of BoNT. Another clinical observation is that BoNT injections may improve muscle tone and function in non-treated body parts, especially in dystonia and spasticity [18]. This has also been proven by numerous electrophysiological studies indicative of BoNT-related effects within non-injected muscles [19,20,21]. In spastic syndromes, evidence for central effects of BoNT comes from the fact that injection in one muscle may also act at the corresponding antagonist muscle [22,23] or other non-injected muscles with consecutive clinical benefit [18,24]. Interestingly, the time course of pain relief differed from that of muscle relaxation in cervical dystonia (CD) following BoNT injections. Improvement in pain occurred before motor improvement, and pain relief was even longer lasting than muscle weakness [25]. These facts further suggest that pain relief could be attributed to additional effects on sensory nerve fibers. Regardless, higher doses were needed to successfully treat CD patients with dystonia-related pain, as compared to pain-free CD patients [26]. However, other authors suggested a muscular mechanism for the genesis of pain in CD [27]. Further evidence for (rather indirect) central effects of BoNT stems from a behavioral study investigating spatial discrimination thresholds in patients with CD before and following BoNT treatment. Here, BoNT injections restored abnormally increased thresholds of spatial discrimination, a clinical marker of disorganized sensory cortical somatotopy in dystonia [28]. This suggests the modulation of afferent inputs to the sensory cortex from muscle spindles [29].

In summary, there is unequivocal clinical evidence for an (indirect) central effect of BoNT, an observation that is further underscored by numerous human electrophysiological studies.

## 3. Neurophysiological Evidence

A large number of clinical neurophysiological studies provide detailed evidence for distinct BoNT effects within spinal cord circuitries, the brainstem and the sensorimotor cortex.

Trompetto et al. recognized that BoNT acts differently on extra- and intrafusal muscle spindles as measured by maximal M-wave and maximal voluntary contraction (both affect extrafusal spindles through synaptic blockade) as well as the tonic vibration reflex (an intrafusal effect through the inhibition of the stretch reflex loop). In this study of patients with focal hand dystonia (FHD), BoNT induced a persistent clinical benefit even though indicators of extrafusal chemodenervation had fully recovered. This suggests that changes in Ia-afferences may have resulted in (indirect) central effects [30]. F-wave reductions have been reported in distant, non-injected muscles of patients with CD following BoNT, an observation that was interpreted by the authors as a potential consequence of reduced spinal motoneuronal excitability [31]. This was experimentally proven in another study that measured recurrent inhibition in distant, non-injected muscles of patients treated for lower limb spasticity. Here, Marchand-Pauvert and colleagues demonstrated the depression of recurrent inhibition from soleus motor axons to motoneurons supplying the quadriceps muscle after BoNT injection in the soleus muscles [32]. The authors concluded that BoNT affects spinal synaptic transmission via its effect on cholinergic synapses of Renshaw cells in humans through retrograde transport.

Brainstem excitability can be experimentally studied using the blink reflex and its variations, or brainstem auditory evoked potentials. Several studies did not reveal any modulation of hyperexcitable brainstem pathways in patients with blepharospasm following BoNT injections into the orbicularis oculi muscle [20,33,34]. Furthermore, BoNT did not affect brainstem auditory-evoked potentials in patients with craniocervial dystonia and hemifacial spasm [35]. On the other hand, BoNT therapy led to reductions in muscle activity of the injected thyroarytenoid muscle, together with the non-injected, contralateral thyroarytenoid muscle in patients with spasmodic dysphonia. This was interpreted as excitability changes at the brainstem level [36]. Another way to study brainstem circuit excitability and plasticity is via a conditioning high-frequency stimulation protocol that may induce long-term potentiation of the human blink reflex [37]. Here, the previously enhanced facilitation of the R2 response of the blink reflex in patients with blepharospasm was normalized by BoNT [38], again suggestive of central effects of BoNT at the brainstem level. However, enhanced plasticity and its normalization following BoNT was not confirmed in a subsequent study [39].

Modulation of Ia-afferences and the stretch reflex loop were considered responsible for the increase in somatosensory evoked potentials following BoNT injection in patients with cerebral palsy [40,41] (see also [42]). In a separate study, contrary effects of BoNT on the somatosensory cortex were observed, as intramuscular injections of BoNT normalized previously abnormally enhanced amplitudes of somatosensory-evoked potentials in patients with CD [43]. Whereas the previous study suggested the normalization of increased excitability of the sensory cortex by BoNT, Gilio and colleagues reported the normalization of both increased motor cortical excitability and reduced intracortical inhibition following BoNT injection [44]. However, the observation of normalized motor cortical excitability following BoNT therapy was not confirmed by subsequent studies [45,46]. Ways to investigate sensorimotor interplay at the cortical level are called long-latency reflexes. Here, two electromyographic responses at the thenar muscle are recorded following median nerve stimulation at different latencies that may reflect the interaction of somatosensory input and motor cortical output at the spinal level (stretch reflex) and via the cortex [47]. Following BoNT treatment, the second cortical (but not the spinal) response was reduced on the affected side in both patients with FHD and CD. These findings point to a modulatory BoNT effect on the afferent input that leads to changes in (previously abnormal) motor cortical overflow (see also [48]). Changes in motor cortical excitability, as probed by measuring motor evoked potentials, can be induced experimentally by a protocol called paired associative stimulation. This combines repetitive electrical stimulation of a hand nerve with subsequent transcranial magnetic stimulation of the contralateral motor cortex [49]. Abnormally enhanced changes in motor cortical excitability following this paired association stimulation protocol in dystonia [50,51] were blocked by BoNT injections in CD patients [52]. This supports the idea of BoNT inducing changes of the afferent input, which in turn results in the blockage of the (previously enhanced) plasticity of the sensorimotor cortex in focal dystonia. Changes in motor cortical reorganization following BoNT therapy have also been reported in other transcranial magnetic stimulation studies. These mapped the topography of the primary motor cortex projections of upper limb muscles in patients with FHD, CD and primary hand tremor [53,54,55]. However, in another study [28], the somatotopic finger and hand muscle representations in the motor cortex were retained in FHD at rest. This implies that abnormal motor organization may only arise during activation, when abnormal somatosensory representations are functionally integrated.

## 4. Evidence from Neuroimaging

While numerous neurophysiological studies provide strong yet indirect evidence for the functional central effects of BoNT, an increasing amount of human neuroimaging studies, predominantly in patients with dystonia and spasticity, indicate distinct functional but also structural brain changes induced by peripheral BoNT injections.

Early imaging studies made use of H_2_^15^O positron emission tomography (PET) in order to investigate possible changes in cortical activation patterns following BoNT therapy. In an early study by Ceballos-Baumann and colleagues in patients with FHD, BoNT improved writing but failed to improve the associated reductions of the regional cerebral blood flow (a marker of neuronal activity) of the primary motor cortex [56]. On the other hand, BoNT treatment resulted in increased neuronal activation of the parietal cortex and secondary motor areas, such as the caudal supplementary motor area. This was interpreted as a change in movement strategy or associated cortical reorganization, secondary to the deafferentation of alpha motor neurons. This interpretation was supported by another study in spasmodic dysphonia patients. It revealed increased speech-related responses in sensory cortical areas and in left hemisphere motor areas commonly associated with oral-laryngeal motor control, following BoNT injections [57]. This study further indicated that BoNT treatment may lead to a more efficient cortical processing of sensory information to relevant motor areas. In addition, unilaterally injected BoNT reduced the primarily increased thalamic activation bilaterally in hemifacial spasm. This suggests that BoNT induced changes of afferent input from the skin and muscle spindle, as well as the antidromic conduction of the facial nerve and secondary alteration in the central nervous system [58].

The largest neuroimaging evidence for the central effects of BoNT comes from functional magnetic resonance imaging (fMRI) studies. Several studies reported a decreased, and in part more lateralized, activation pattern in contra- and ipsilateral cortical motor areas following BoNT therapy in patients with spastic hemiparesis due to stroke [59,60,61]. However, apart from the primary sensorimotor cortex, changes of activation patterns were also found in other brain areas such as the cerebellum [60,61,62,63], the supplementary motor area [60] and the parietal and occipital cortex [61,64]. These findings point to BoNT-induced changes in cortical reorganization that may, in addition to the peripheral BoNT effect, result in the relief of spasticity and a better motor function, possibly due to a more focused activation of stroke-affected areas. 

In dystonia, cerebral activation patterns are not confounded by stroke-related parenchymal injury. Here, BoNT treatment was associated with widespread changes on fMRI-measured activation (i.e., BOLD signal) within several brain regions, such as the bilateral primary and secondary somatosensory cortex, the bilateral supplementary motor area, the contralateral primary motor cortex and the cerebellum in drug-naïve CD patients [65]. Hence, only the first BoNT injection session resulted in changes to sensorimotor activation patterns. Interestingly, no differences in activation patterns between patients following BoNT treatment and healthy controls at baseline were observed. Several previous studies investigating orofacial dystonia [66,67] or CD [68,69] showed significant changes within the sensorimotor network in patients receiving long-term treatment with BoNT, in comparison to healthy controls. Despite one study on patients with spasmodic dysphonia [70], changes in brain activation pattern were also demonstrated four weeks after BoNT injections. In summary, these studies underline the short- and long-term central effect of BoNT on the fMRI activation pattern in focal dystonia. Furthermore, long-term effects of BoNT therapy have to be considered when comparing brain activation patterns in patients with dystonia and healthy controls.

Resting-state fMRI allows the investigation of distinct brain networks and the interaction between different brain areas at rest [71]. This possibly overcomes the fact that cortical activation in some previous fMRI studies on dystonia may be confounded by (dystonic) movements. Interestingly, impaired functional resting state connectivity within the sensorimotor and basal ganglia network was found in focal hand dystonia [72,73,74], task-specific orofacial dystonia [75], blepharospasm [76] and CD [77,78]. However, it remains unknown whether these alterations are primary or secondary, perhaps compensatory phenomena. In some studies, BoNT treatment could at least in part modulate disease-related altered functional connectivity patterns within specific brain regions in focal dystonia [76,77,78] (see also [72]). This is probably again done by altering sensory input.

**Figure 1:**
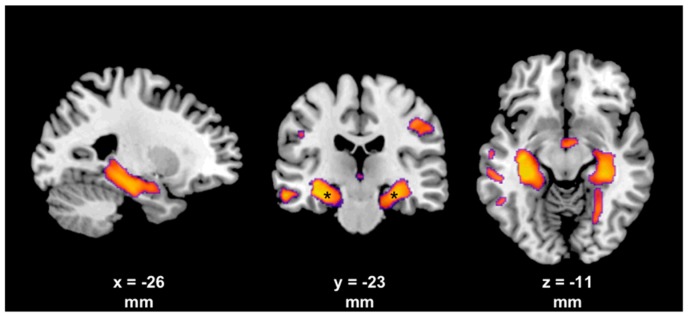
T-score maps showing a smaller gray matter volume in long-term BoNT-treated CD patients compared to untreated CD patients. Results are illustrated at an exploratory threshold (voxel-wise *p* < 0.005, cluster size >100 voxel; * *p* < 0.05, FWE whole brain corrected on the cluster level) with the corresponding location within the MNI space indicated below.

Few studies exist that explicitly investigated structural changes of the brain due to BoNT therapy in focal dystonia patients. Two studies found white matter abnormalities in the basal ganglia regions in patients with CD and FHD, using diffusion tensor imaging [79,80]. These ultrastructural changes were normalized four weeks following BoNT injections. This observation was interpreted as (preliminary) evidence for activity-dependent brain white matter plasticity due to indirect effects on motor afferent feedback to brain motor regions. These included the thalamus, the sensorimotor cortex and, indirectly, the basal ganglia [79]. Widespread gray matter changes were found in different forms of focal dystonia using different volumetric techniques (i.e., voxal-based morphometry, cortical thickness). These changes occurred in the motor and premotor cortex, the cerebellum, the basal ganglia, the thalamus and the parietal cortex [81,82,83,84,85,86,87]. Only a few studies investigated the short-term effect of BoNT [88,89,90]. Delnooz and colleagues reported an increase in gray matter volume (GMV) exclusively within the right precentral sulcus following BoNT treatment in patients with CD. This indicated indirect central consequences of modified peripheral sensory input [90]. In another study, BoNT therapy resulted in substantial cortical thickness reductions within the primary motor cortex and the pre-supplementary motor area in patients with blepharospasm, whereas in patients with hemifacial spasm no longitudinal changes were found [89]. Hence, the latter study demonstrated not only BoNT-dependent structural changes but also disease-dependent structural changes of cortical morphology. These findings suggest that the peripheral BoNT effect may not be solely responsible for the (indirect) central effect of BoNT. We recently compared the short- and long-term effect of BoNT in drug-naïve and BoNT-treated CD patients, in order to differentiate disease- and therapy-specific gray matter changes (Figure 1, [88]). Interestingly, both groups only differed in bilateral mesiotemporal GMV, suggesting long-term effects of continuous BoNT therapy. Alternatively, disease duration may be responsible for these reductions in hippocampal GMV, or may at least have confounded our findings.

## 5. Limitations

All the studies presented here used BoNT type A. Hence, no conclusions can be made regarding other types of BoNT such as type B, which is also commercially used. However, as all types of BoNT act at the cholinergic presynapse, blocking acetylcholine release at the neuromuscular junction, the indirect central effect should not significantly differ between different types of BoNT. Furthermore, patients were investigated in a clinical setting, not reporting or differentiating between dosage, injected muscles or time since last BoNT injection. However, for baseline investigations, patients were studied at least 12 weeks since their last BoNT treatment, when the clinical effect of BoNT should have been minimized. They were usually investigated around 4 weeks following BoNT injections. However, as reported in our last study [88], drug-naïve and BoNT-treated patients differed regarding gray matter changes. This suggested that the short-term effects of BoNT may have been confounded by long-lasting BoNT effects on the CNS in previous studies. Additionally, 12 weeks following the last BoNT injection may not be enough to exclude remaining central BoNT effects, especially since we know that the effect on the intrafusal muscle spindles may last longer than on the extrafusal muscles spindles [30].

## 6. Conclusions

BoNT primarily acts at the neuromuscular junction, resulting in a biochemical denervation and muscle weakness of the injected muscle, a mechanism which undoubtedly constitutes the main action and cause for the reliable clinical effect of BoNT in several neurological disorders. Nevertheless, beside its peripheral action, strong clinical, neurophysiological and neuroimaging evidence exists indicating additional BoNT-related central effects. It is somewhat limiting, however, that all these latter investigations were not sufficiently capable to differentiate between direct actions of BoNT on the CNS and indirect effects due to the modulation of the afferent sensory input to the spinal cord and brain. However, the current literature suggests that indirect effects of BoNT on the brain may be more prominent. Here, changes to the afferent input are thought to result in short- and long-term plastic changes to the CNS, as assessed by functional and structural methods. This modulation or reorganization of the brain may itself have an additional therapeutic effect. It may potentially be responsible for the long-lasting clinical effect of BoNT or its effect in non-treated muscles. It will be interesting to see whether other neurophysiological methods such as transcranial magnetic or direct current stimulation will provide novel insight into BoNT’s plasticity effects and whether their clinical application may help to improve and prolong the (positive) central plastic effects.
